# Pregnant, miserable, and starving in 21st century America

**DOI:** 10.1016/j.xagr.2022.100141

**Published:** 2022-12-05

**Authors:** Marlena S. Fejzo, Kimber W. MacGibbon, Katherine L. Wisner

**Affiliations:** 1 From the Department of Maternal-Fetal Medicine, Keck School of Medicine, University of Southern California, Los Angeles, CA (Dr Fejzo); 2 Hyperemesis Education and Research Foundation, Clackamas, OR (Ms MacGibbon); 3 Asher Center for the Study and Treatment of Depressive Disorders, Departments of Psychiatry and Behavioral Sciences and Obstetrics and Gynecology, Northwestern University Feinberg School of Medicine, Chicago, IL (Dr Wisner)

**Keywords:** hyperemesis gravidarum, nausea, pregnancy, vomiting

## Abstract

Severe nausea and vomiting of pregnancy is too common and devastating to be trivialized any longer. Authors of recent studies observed that children exposed in utero to severe nausea and vomiting of pregnancy had an increased risk for autism spectrum disorder, a decreased brain cortical volume, and developmental deficits. Research on severe nausea and vomiting of pregnancy and hyperemesis gravidarum has been disturbingly slow. It was not until 2021 that an international consensus definition was published. Hyperemesis gravidarum starts before 16 weeks’ gestation, is characterized by severe nausea with or without vomiting and an inability to eat and drink normally, and greatly limits daily activities. Maternal misery is caused by unrelenting nausea, intractable retching or vomiting, ptyalism, dehydration, reflux, malnutrition, and social isolation. Hyperemesis gravidarum is the second most common reason for hospitalization in pregnancy. Symptoms can persist until delivery in one-third of individuals who experience extreme weight loss. Significant associations have been identified between hyperemesis gravidarum and multiple adverse outcomes. Maternal deaths owing to hyperemesis gravidarum continue to be reported, and hyperemesis gravidarum is associated with high fetal loss and termination rates. These grim findings highlight the critical public health importance of treating severe nausea and vomiting of pregnancy early to mitigate serious complications that compromise maternal and offspring health during pregnancy and beyond. Despite suffering extreme debility, individuals with hyperemesis gravidarum report feeling that their experiences were dismissed by healthcare professionals, contributing to therapeutic termination, suicidal ideation, perinatal depression, and posttraumatic stress disorder. Hyperemesis gravidarum must be recognized early and treated aggressively with frequent monitoring. Although medications can be effective in reducing symptoms, many patients do not gain adequate relief, and new treatments are needed. A promising new avenue for treatment comes from genetic discoveries. The gene, growth differentiation factor-15, which codes for a nausea and vomiting hormone produced by the placenta, is the greatest genetic risk factor for hyperemesis gravidarum, and therapies are currently in clinical trials in cancer. However, until treatment is universally effective, abortion access must be available for refractory hyperemesis gravidarum. Herein, we emphasize data published since the most recent American College of Obstetrics and Gynecology report (2018), such as long-term neuropsychiatric consequences in offspring exposed to hyperemesis gravidarum and suggest interventions anticipated to prevent progression of early symptoms to hyperemesis gravidarum.

## Introduction

Severe nausea and vomiting of pregnancy (sNVP) is too common and devastating to be trivialized any longer.^1^ Authors of a recent study including 10,710 pregnancies reported that 14% of participants were affected by vomiting that led to maternal weight loss and/or that extended beyond the sixth gestational month. They also observed that children exposed in utero to sNVP had decreased brain cortical volume, which mediated the relationship between sNVP and developmental deficits in language, learning, and motor skills. There is a wealth of data about the importance of stress, depression, and anxiety on the long-term developmental outcomes of children, and the combination of disease-related stress with maternal nutritional deficiencies (which also increase the likelihood of poor mental health) is a potent adverse exposure.^2^ Thus, it is likely that the underlying pathologic mechanisms include maternal nutritional deficiencies and chronic overwhelming stress. Moreover, authors of a recently published retrospective cohort study reported that offspring exposed in utero to hyperemesis gravidarum (HG) had a 53% increased risk for autism spectrum disorder compared with unexposed children. Importantly, they observed that antiemetic medications did not contribute to the risk. Research on sNVP and HG has been disturbingly slow. It was not until 2021 that an international consensus definition was published.^3^ HG starts before 16 weeks’ gestation, is characterized by severe nausea and/or vomiting and the inability to eat and/or drink normally, and greatly limits daily activities. Maternal misery is caused by unrelenting nausea, intractable retching or vomiting, ptyalism, dehydration, reflux, malnutrition, and social isolation. HG is the most common reason for hospitalization in the first half of pregnancy and the second most common throughout pregnancy. Symptoms can persist until delivery in as many as one-third of individuals who experience extreme weight loss (>15% of prepregnancy weight).^1^

Significant associations have been identified between HG and multiple adverse outcomes, including an increased risk estimated to be as high as 3.86-fold greater for preeclampsia, 1.94-fold greater for venous thromboembolism, 1.28-fold greater for anemia, 2.8-fold greater for low birthweight, 4-fold greater for preterm birth, and 5-fold greater for small for gestational age (SGA) neonate.^4^, ^5^, ^6^, ^7^, ^8^ HG is associated with a greater risk for SGA neonates than exposure to chronic hypertension, pregestational diabetes, preeclampsia, autoimmune disease, cocaine, amphetamines, cannabis, and tobacco.^8^ Maternal deaths owing to HG continue to be reported in the United States and the United Kingdom,^1^ and HG is associated with high fetal loss and termination rates.^1^

These grim findings highlight the critical public health importance of treating sNVP early to mitigate serious complications that compromise maternal and offspring health during pregnancy and beyond. Despite suffering extreme debility, individuals with HG report feeling that their experiences of sNVP were dismissed by healthcare professionals, contributing to a substantial incidence of therapeutic termination, suicidal ideation, perinatal depression, and posttraumatic stress disorder.^4^^,^^9^ Emotional distress is secondary to the profound misery caused by HG, although erroneously presumed to cause HG.

HG must be recognized early and treated aggressively with frequent monitoring. However, pregnant individuals and their clinicians are often reluctant to use medication despite the risks for worsening symptoms and complications. The thalidomide tragedy (in which babies were born with major limb deformities to mothers who took thalidomide for HG) has led to the overestimation of the teratogenic risk of many medications used in pregnancy. In addition, fear of litigation has discouraged the appropriate testing of drugs used during pregnancy, leading to a lack of evidence and/or transparency on the safety and effectiveness.^10^ Although medications can be effective in reducing symptoms, many patients do not gain adequate relief, and new treatments are urgently needed. A promising new avenue for treatment comes from genetic discoveries.^4^ The gene, growth differentiation factor-15 (GDF15), which codes for a nausea and vomiting hormone produced by the placenta, was discovered to be the greatest genetic risk factor for HG. GDF15 also drives cancer cachexia, a disease with similar symptoms to HG. Therapies targeting the GDF15 pathway are currently in clinical trials and may provide a novel therapy for conditions similar to cachexia, such as HG. But until treatment is universally effective, abortion access must be available for refractory HG associated with maternal morbidity and mortality.

Treatment guidelines are available from the American College of Obstetrics and Gynecology. In this clinical opinion, we emphasize data published since their most recent report (2018), such as long-term neuropsychiatric consequences in offspring exposed to HG, and suggest interventions anticipated to prevent progression of early symptoms to HG.

## Early identification

Symptoms may begin before 6 weeks’ gestation and before the first obstetrical contact. Patients may present to the emergency room or primary care clinicians for evaluation before pregnancy recognition. At a minimum, patients at increased risk for HG (previous HG pregnancy and/or family history of HG) should be monitored early in pregnancy and treated proactively. Because NVP is common, patients and practitioners may accept the experience of a substantial burden of disease before initiating treatment. Intervening before symptoms intensify with oral or nonoral antiemetic medications and intravenous hydration is optimal.

## Increase awareness of the complications of severe nausea and vomiting of pregnancy and hyperemesis gravidarum

Updated education on new findings about the pathogenesis and adverse outcomes will address the outdated views that sNVP has a psychological etiology and does not affect offspring development. Emergency department contacts for HG are rising and account for almost 400,000 visits in the United States annually. Increased education for emergency and obstetrical clinicians on treatment guidelines in addition to the new findings provides a critical structure for improving care.

## Provide resources for the pregnant people and their support system

Connecting patients and advocacy organizations adds to the support available to this sizable group of affected individuals. The Hyperemesis Education and Research Foundation has numerous resources for providers, patients, and their families (www.hyperemesis.org/).

## Routinely prescribe oral and intravenous vitamins for all patients with severe nausea and vomiting of pregnancy

Because nutritional deficiency is a hallmark of HG and contributes to adverse outcomes, supplemental vitamins and/or nutrition is imperative; however, oral administration is often not tolerated. Patients should be monitored to determine whether supplements or intravenous vitamins are required. Patients with HG are at increased risk for Wernicke's encephalopathy because of a thiamine (vitamin B1) deficiency after a few weeks of sNVP. Initial symptoms include ocular changes, ataxia, and confusion, which may progress to profound cardiac and neurologic sequalae. Offspring are at increased risk for neural tube defects and embryopathy secondary to folate and vitamin K deficiency, respectively.

## Refer patients for supportive care

As sNVP improves, patients must be evaluated for posttraumatic stress disorder. Interventions such as trauma-informed psychotherapy, nutritional consultation, and physical therapy may be necessary. HG has long-term consequences that require extended management of multiple physical and psychological symptoms. Of note, it was recently found to be one of the greatest risk factors for postpartum depression.^11^

In the United States, approximately half a million pregnancies are affected by sNVP and are at risk for associated persistent adverse maternal and child outcomes. Thus, an immediate call to action for the identification, early intervention, and aggressive treatment of sNVP is compelling. These constructive starting points are critical to improving obstetrical, emergency, and pediatric healthcare and lowering adverse maternal and fetal outcomes, including therapeutic termination.

## References

[1] Fejzo MS, Mullin P., Martin CR, Preedy VR, Rajendram R (2021). Diagnosis, management and modeling of neurodevelopmental disorders.

[2] Monk C, Fernández CR. (2022). Neuroscience advances and the developmental origins of health and disease research. JAMA Netw Open.

[3] Jansen LAW, Koot MH, Van't Hooft J (2021). The Windsor definition for hyperemesis gravidarum: a multistakeholder international consensus definition. Eur J Obstet Gynecol Reprod Biol.

[4] Fejzo MS, Trovik J, Grooten IJ (2019). Nausea and vomiting of pregnancy and hyperemesis gravidarum. Nat Rev Dis Primers.

[5] Nurmiaty, Asi M, Aisa S, Halijah, Yustiari, Usman AN. (2021). Eating habits and history of hyperemesis gravidarum as a risk factor of preeclampsia. Gac Sanit.

[6] Fiaschi L, Nelson-Piercy C, Gibson J, Szatkowski L, Tata LJ. (2018). Adverse maternal and birth outcomes in women admitted to hospital for hyperemesis gravidarum: a population-based cohort study. Paediatr Perinat Epidemiol.

[7] Fejzo MS, Magtira A, Schoenberg FP (2013). Antihistamines and other prognostic factors for adverse outcome in hyperemesis gravidarum. Eur J Obstet Gynecol Reprod Biol.

[8] Sasso EB, Bolshakova M, Bogumil D (2021). Marijuana use and perinatal outcomes in obstetric patients at a safety net hospital. Eur J Obstet Gynecol Reprod Biol.

[9] Nana M, Tydeman F, Bevan G (2021). Hyperemesis gravidarum is associated with increased rates of termination of pregnancy and suicidal ideation: results from a survey completed by >5000 participants. Am J Obstet Gynecol.

[10] Goodwin TM, Poursharif B, Korst LM, MacGibbon KW, Romero R, Fejzo MS. (2008). Secular trends in the treatment of hyperemesis gravidarum. Am J Perinatol.

[11] Munk-Olsen T, Liu X, Madsen KB (2022). Postpartum depression: a developed and validated model predicting individual risk in new mothers. Transl Psychiatry.

